# Thrombus in the Right Coronary Sinus of Valsalva Originating From the Left Atrial Appendage Causing Embolic Inferior Wall Myocardial Infarction

**DOI:** 10.1177/2324709618792023

**Published:** 2018-07-24

**Authors:** Hossam Abubakar, Ahmed S. Ahmed, Ahmed Subahi, Ahmed S. Yassin

**Affiliations:** 1 Wayne state University, Detroit, MI, USA; 2 St. Bernard Heart and Vascular, Jonesboro, AR, USA

**Keywords:** aortic root thrombus, left atrial appendage thrombus, embolic myocardial infarction

## Abstract

Acute myocardial infarction (MI) is commonly a result of coronary atherosclerotic plaque rupture and superimposed thrombus formation. Nevertheless, uncommon causes of MI including embolism from aortic root and ascending aorta mural thrombi must be considered when coronary atherosclerotic disease is not evident. We report a case of a 84-year-old woman who presented with an inferior ST-segment elevation MI. Initial attempts to engage the right coronary artery (RCA) were unsuccessful. Aortic angiography revealed evidence of the left coronary artery ostium with absence of the right coronary ostium or RCA. Probing with a coronary wire where the RCA ostium was presumed to be located yielded resolution of the ST-segment elevation. The RCA was then easily engaged using a guide catheter, and angiographic evaluation showed a smooth vessel with no evidence of coronary artery disease except for abrupt termination of the distal PL2 branch. Contrast-enhanced computed tomography revealed an aortic root thrombus extending into the right coronary sinus of Valsalva and a thrombus in the left atrial appendage. The case reveals RCA embolism from an aortic root thrombus likely originating from the left trial appendage. A conservative approach to treatment with anticoagulation was pursued that resulted in full recovery. A review of the literature revealed that the etiology of aortic root thrombi is proposed to be multifactorial. Prospective randomized studies are needed to demonstrate the best treatment approach, although this appears to be impracticable given the rarity of the disease.

## Introduction

Acute myocardial infarction (MI) is commonly a result of coronary atherosclerotic plaque rupture and superimposed thrombus formation. Nevertheless, uncommon causes of MI including embolism from aortic root and ascending aorta mural thrombi must be considered when coronary atherosclerotic disease is not evident. The mechanism behind aortic root thrombus formation remains unclear and is likely multifactorial incorporating all 3 components of the Virchow’s triad. This report presents a rare case of acute MI secondary to a thrombus in the right coronary sinus of Valsalva possibly originating from the left atrial appendage, successfully treated with anticoagulation. We reviewed the literature for similar cases and discussed the possible etiopathogeneses and different treatment modalities for this rare entity.

## Case Presentation

**Figure 1.**
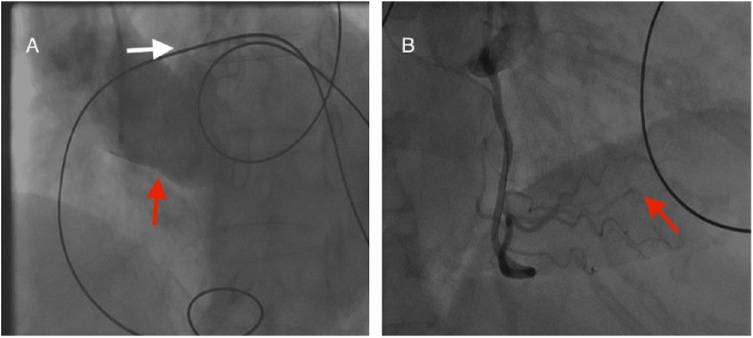
Aortic root angiogram showing evidence of the left main coronary artery originating from the left coronary artery ostium (white arrow) with absence of the right coronary ostium or right coronary artery (red arrow; A) and a right anterior oblique view of the right coronary artery showing no angiographic evidence of coronary artery disease with abrupt occlusion of the distal posterolateral branch (PL2, red arrow; B).

**Figure 2.**
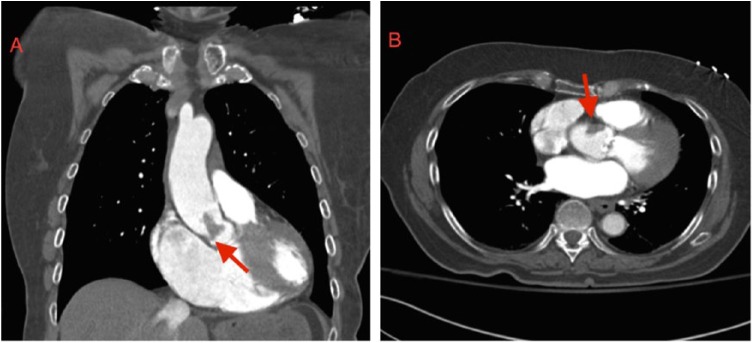
Contrast-enhanced computed tomography revealed the presence of an aortic root mass 21 × 16 mm suggestive of a thrombus, extending into the right coronary sinus of Valsalva (red arrow; A) with near complete obliteration of the left atrial appendage with another large thrombus (red arrow; B).

An 84-year-old woman with hypertension presented to the emergency department with epigastric pain, nausea, and dizziness for 3 hours. A 12-lead electrocardiogram showed a junctional rhythm at rate of 40 and 2 mm inferior ST-elevations with lateral ST depressions. High-sensitivity troponin-I level was 0.01 ng/mL. Initial management included aspirin, clopidogrel, and intravenous heparin, and she was subsequently taken emergently to the catheterization laboratory. Attempts to engage the right coronary artery (RCA) were unsuccessful despite using multiple guide catheters. The left coronary system showed no angiographic evidence of coronary artery disease with left to right collaterals. Contrast injection in the right coronary sinus suggested ostial total occlusion of the RCA (Figure 1A). Probing with a coronary wire near where the RCA ostium was presumed to be located was associated with an increase in the heart rate with an idioventricular rhythm and resolution of inferior ST-elevation. The RCA was then easily engaged with a guide catheter. Angiographic evaluation of the RCA showed a smooth vessel with no evidence of coronary artery disease except for abrupt termination of the distal PL2 branch (Figure 1B). A computed tomography angiogram was then done to explore the cause of the right ostial occlusion and revealed an aortic root thrombus (21 × 16 mm) with extension into the right coronary sinus, together with near complete obliteration of the left atrial appendage with another large thrombus (Figure 2A and B). Serial electrocardiograms demonstrated paroxysmal atrial fibrillation with complete resolution of inferior ST-segment elevation. Subsequent troponin-I levels peaked at 74 ng/mL. A transthoracic echocardiogram showed inferobasal septal hypokinesis and ejection fraction of 45%. A brain magnetic resonance imaging obtained secondary to mental status changes that occurred a few hours after the procedure showed multiple embolic cerebral infarcts and complete occlusion of the left internal carotid artery. The patient was treated with intravenous heparin and bridged to warfarin therapy. She was discharged home in good condition on hospital day 5. Follow-up 6 months after the index hospitalization revealed no symptoms or signs of disease recurrence.

## Discussion

Although atherosclerotic coronary artery disease is the major cause of acute coronary syndrome, thromboembolism remains a well-recognized etiology for MI.^1^ Mural thrombosis of the aorta is well described in patients with clinical evidence of thromboembolic disease and also in asymptomatic patients. The majority of these cases are associated with aneurysmal disease with nonaneurysmal mural thrombosis of the ascending aorta being an exceedingly rare entity.^2^ A large postmortem study evaluating 10 671 autopsy specimens showed a 0.4% incidence of nonaneurysmal thoracoabdominal aortic mural thrombosis, with only 0.1% of cases involving the ascending aorta.^2^ While systemic embolism seems to be the main determinant of morbidity and mortality in this uncommon disease, data on coronary artery occlusion secondary to aortic thrombi is limited to case reports and case series. We report a rare case of nonaneurysmal aortic root thrombus originating from the left atrial appendage extending into the right coronary sinus of Valsalva causing complete ostial occlusion of the RCA presenting with acute MI. A PubMed search identified 16 similar cases of aortic root thrombi causing RCA embolic MI (Table 1).

**Table 1.** Reported Cases of Aortic Root Thrombi Causing Right Coronary Artery (RCA) Occlusion.

Author/Year	Age/Gender	Risk Factors	Thrombus Size/Location	Method of Diagnosis	Clinical Presentation	Non–Coronary Embolization	Aortic Wall Pathology	Treatment	Outcome
Ennezat et al^3^ (2006)	60/Male	Prosthetic aortic valve	Non–coronary sinus of Valsalva	TEE	Inferolateral MI	Cerebral embolism	Not reported	Aspirin, heparin	Uneventful survival
	69/Male	Prosthetic aortic valve	Right coronary sinus of Valsalva	TEE	Inferolateral MI	Cerebral embolism	Not reported	Aspirin, warfarin	Uneventful survival
		HIT							
Knoess et al^4^ (2007)	30/Female	Smoking	8 × 20 cm	Autopsy	Weakness, dizziness, and sudden death	Absent	Absent	Absent	Death
		DM	1 cm above the RCA ostium						
		Pregnancy							
Mizuguchi et al^5^ (1994)	78/Female	AF	Right coronary sinus of Valsalva	MDCT	Inferior MI	Absent	Not reported	Catheter thrombus aspiration	Uneventful survival
		Protein C and S deficiency							
Nakamori et al^6^ (2009)	78/Female	Absent	40 × 30 mm	Contrast-enhanced CT	Inferior MI	Absent	Absent	Surgical thrombectomy	Uneventful survival
			Right coronary sinus of Valsalva						
Papachristidis et al^7^ (2016)	20/Male	MDMA	11 × 7 mm	TEE	MI	Absent	Not reported	Surgical thrombectomy	Uneventful survival
			Sino-tubular junction						
Saygi et al^8^ (2011)	46/Male	Heterozygote polymorphism of MTHFR C677T	25 × 10 mm	TEE	MI	Absent	Aortic wall erosion	Surgical thromboembolectomy	Uneventful survival
		Homozygote polymorphism of PAI 1 4G/5G	Non–coronary sinus of Valsalva						
Tamura et al^9^ (2011)	59/Male	Smoking	Right coronary sinus of Valsalva	CECT	Inferior MI	Absent	Aortic wall erosion	Surgical thrombectomy	Uneventful survival
				TEE					
Nishizaki et al^10^ (2003)	49/Female	Smoking	Ascending aorta	CT	Inferior MI	Renal artery	Erosion of atheromatous plaque	Surgical thrombectomy	Uneventful survival
		HLD							
		COCPs							
Bertrand et al^11^ (2009)	61/Male	Not reported	Ascending aorta above the RCA ostium	Left anterior oblique ventriculography	Inferior MI	Absent	Ulcerated atheromatous plaque	Surgical thrombectomy	Uneventful survival
Eguchi et al^12^ (2004)	56/Male	Smoking	18 × 4 mm	TEE	Inferolateral MI	Absent	Aortic wall erosion	Surgical thrombectomy	Uneventful survival
		HLD	Ascending aorta above the RCA ostium						
		Protein S deficiency							
Shahin et al^13^ (2002)	37/Female	Smoking	RCA with extension to the aorta from the RCA ostium	TEE	Inferior MI	Absent	Not reported	Surgical thrombectomy	Uneventful survival
									
Christiaens et al^14^ (1996)	41/Male	HTN	10 mm	TEE	Inferior MI	Limb ischemia	Absent	Surgical thrombectomy	Uneventful survival
			Non–coronary sinus of Valsalva						
Decker et al^15^ (1995)	Female (age not reported)	Not reported	Ascending aorta	TEE	MI (anatomy not specified)	Absent	Absent	Heparin	Uneventful survival
Dik et al^16^ (1993)	46/Female	Smoking	Ascending aorta near the RCA ostium	TEE	Inferior MI	Absent	Aortic wall erosions	Surgical thrombectomy	Uneventful survival
		Progesterone therapy							

Abbreviations: AF, atrial fibrillation; CECT, contrast-enhanced computed tomography; COCPs, combined oral contraceptive pills; CT, computed tomography; DM, diabetes mellitus; HIT, heparin-induced thrombocytopenia; HLD, high-density lipoprotein; HTN, hypertension; MDCT, multidetector computed tomography; MDMA, methylenedioxymethamphetamine; MI, myocardial infarction; TEE, transesophageal echocardiography.

The etiology of thrombus formation in the aorta remains unclear and is likely multifactorial, incorporating all 3 arms of the Virchow’s triad (blood stasis, hypercoagulable state, and endothelial injury). First, the aorta is a site of high blood flow velocity that precludes blood stasis and thrombus formation. Nevertheless, the aortic sinuses instigate the development of eddy currents that function in holding the aortic valve leaflets away from the aortic wall aiding in their closure during systole.^17^ These currents create blood stasis and turbulence increasing the likelihood of thrombus formation in the aortic sinuses.^18^ Furthermore, endothelial injury is also a likely culprit. In 7 of the 16 previously described cases, aortic wall abnormalities were noted. Bertrand et al^11^ described a ruptured atherosclerotic plaque while the remaining 6 cases identified superficial erosive lesions. Although the current paradigm sates that plaque rupture is a prerequisite to thrombus formation,^19^ it was shown that superficial endothelial erosions with an intact nonruptured fibrous cap may lead to superimposed thrombus formation.^20^ This observation was found to be more common in women with prothrombotic risk factors including smoking, diabetes, and hyperlipidemia,^20^ which were evident in the majority (5/6) of our reviewed cases with aortic plaque erosions. The last arm of the Virchow’s triad—hypercoagulable state—also plays a role and was evident in 9 of the reported cases. Risk factors reported included prosthetic aortic valves,^3^ heparin-induced thrombocytopenia,^3^ protein S deficiency,^12^ and estrogen and progesterone hormonal therapy.^10,16^ Ennezat et al^3^ and Eguchi et al^12^ reported multiple thrombi attached to different sites of the ascending aorta and aortic arch, which favors the hypercoagulable state as a contributor to thrombus formation rather than local aortic wall atherosclerotic disease. In the cases by Decker et al^15^ and Shahin et al,^13^ no risk factors for hypercoagulable state or aortic wall disease were identified. The authors proposed retrograde thrombus migration from the RCA to the aorta. The only reported case that reported atrial thrombosis as the source of aortic root thrombosis was that by Mizuguchi et al.^5^ Similarly, we suspect that the source of aortic thrombus in our patient is likely the left atrial appendage. However, we feel that other factors including aortic wall endothelial injury cannot be excluded as contributors on the basis of noninvasive diagnostic studies.

Appropriate treatment of thrombi in the ascending aorta remains unclear and multiple modalities of treatment have been described including anticoagulation, thrombolysis,^21^ catheter thromboembolectomy,^13^ and surgical thromboembolectomy.^22^ Most reported cases of RCA occlusion secondary to aortic root thrombus were treated with surgical thromboembolectomy. The rationale underlying an invasive approach lies behind the risk of recurrent embolism associated with conservative treatment with anticoagulation. In a study by Laperche et al, 15 patients with thrombi in the aortic arch were treated with long-term anticoagulation and 27% suffered recurrent embolism.^22^ In a meta-analysis of case reports and case series conducted by Fayad et al, surgical intervention was found to be associated with better outcomes when compared with anticoagulation therapy for nonaneurysmal, nonatherosclerotic mural thrombi.^23^ Due to our patient’s elderly age and the considerable risk of mortality associated with cardiac surgery following acute MI,^24^ we decided to pursue medical management. Our case is one of a few that demonstrated success of conservative medical management with anticoagulation^3,15^ in patients with RCA occlusion secondary to aortic thrombi.

## Conclusion

Acute MI with absence of coronary atherosclerotic disease should warrant the consideration of embolic disease complicating aortic root thrombosis. This carries great importance, as treatment may be substantially different than that of conventional atherosclerotic coronary artery occlusion. Prospective randomized studies are needed to demonstrate the best treatment approach, although this appears to be impracticable given the rarity of the disease.
